# Variations in biomarkers of dyslipidemia and dysbiosis during the menstrual cycle: a pilot study in healthy volunteers

**DOI:** 10.1186/s12905-021-01306-4

**Published:** 2021-04-20

**Authors:** Helena Bergström, Lena Ekström, Anna Warnqvist, Peter Bergman, Linda Björkhem-Bergman

**Affiliations:** 1 Division of Clinical Geriatrics, Department of Neurobiology, Care Sciences and Society (NVS), Karolinska Institute, Blickagången 16, Neo floor 7, 141 83 Huddinge, Sweden; 2 Division of Clinical Pharmacology, Department of Laboratory Medicine, Karolinska Institutet, and Karolinska University Laboratory, Karolinska University Hospital, 141 83 Huddinge, Sweden; 3 Division of Biostatistics, Department of Environmental Medicine, Karolinska Institutet, Nobels väg 13, 171 77 Stockholm, Sweden; 4 Division of Clinical Microbiology, Department of Laboratory Medicine, Karolinska Institutet, ANA Futura, Alfred Nobels Allé 8, 141 52 Huddinge, Sweden; 5 Department of Infectious Diseases, Immunodeficiency Unit, Karolinska University Hospital, 141 83 Huddinge, Sweden; 6 Stockholms Sjukhem, Palliative Medicine, Mariebergsgatan 22, 112 19 Stockholm, Sweden

**Keywords:** Cardiovascular disease, Atherosclerosis, Menstrual cycle, Dyslipidemia, Apolipoprotein B (ApoB), Non-High-Density Lipoprotein-Cholesterol (non-HDL-C), Remnant-Cholesterol (remnant-C), Dysbiosis, Gut microbiota, Trimethylamine N-oxide (TMAO)

## Abstract

**Background:**

Dyslipidemia in metabolic syndrome may introduce an underestimation of the risk for cardiovascular disease (CVD) using Low-Density Lipoprotein-Cholesterol (LDL-C) as a surrogate marker. Recently, non-High-Density Lipoprotein-Cholesterol (non-HDL-C), Apolipoprotein B (ApoB) and remnant-Cholesterol (remnant-C) have been suggested as better biomarkers for dyslipidemia. In addition, the microbial metabolites trimethylamine-N-oxide (TMAO), betaine and choline have been associated with CVD and suggested as markers for dysbiosis. There is a lack of knowledge on potential alterations in these biomarkers during the menstrual cycle. The aim of this single center, prospective non-interventional study, was to investigate variations in biomarkers of dyslipidemia and dysbiosis in healthy volunteers during the menstrual cycle.

**Method:**

Serum samples were collected from 17 healthy, regularly menstruating women during two menstrual cycles, including the follicular, ovulatory and luteal phases. Levels of lipoproteins, lipoprotein ratios and microbial metabolites were analyzed in a total of 90 samples (30 complete menstrual cycles).

**Results:**

ApoB, ApoB/HDL and non-HDL-C/HDL ratios were significantly higher in the follicular phase compared to the ovulatory and luteal phases (*p* < 0.05). Remnant-C were higher during the luteal phase (*p* < 0.05). TMAO did not vary during the different phases and did not correlate with estrogen levels.

**Conclusion:**

Our data support that biomarkers for dyslipidemia vary during the menstrual cycle. Thus, to avoid an underestimation of cardiovascular risk, sampling during the follicular phase, when levels of pro-atherogenic lipids are higher, may be considered.

## Background

During the last decades, there has been a steady rise in the prevalence of the cardiometabolic risk factors known as metabolic syndrome (MetS) [1–3]. Globally, obesity (BMI > 30 kg/m^2^) is now more common in women than men (15% vs 11%). Concomitantly, while the prevalence of acute myocardial infarction (AMI) has decreased in men aged 35–54 years, it has increased in women in midlife [1, 4].

Not surprisingly, there are also sex differences in gut microbiota [5] and in the age-adjusted prevalence of MetS. In addition, dysbiosis, an imbalance of the gut bacteria, has been associated with the MetS and with estrogen homeostasis [6–8].

The association between MetS and the increased risk for atherosclerosis, cardiovascular disease (CVD) and type 2 diabetes are well known [2, 3, 9]. Furthermore, the triad of dyslipidemia i.e. hypertriglyceridemia, increased Low-Density Lipoprotein-Cholesterol (LDL-C) and decreased High-Density Lipoprotein-Cholesterol (HDL-C) observed in MetS, introduces an underestimation of the risk for cardiovascular disease (CVD) using only LDL-C as a surrogate marker [10].

Recently, non-High-Density Lipoprotein-Cholesterol (non-HDL-C) and/or Apolipoprotein B (ApoB) were added as new biomarkers for CVD risk assessment according to guidelines from European Society of Cardiology (ESC) and European Society of Atherosclerosis (EAS) [11]. The latest biomarker to be introduced is remnant-Cholesterol (remnant-C), which includes the cholesterol in Triglyceride (TG)-Rich Lipoproteins (TGRLs)-Very Low-Density lipoprotein (VLDL), Intermediate-Density Lipoprotein (IDL) and Chylomicron remnant [12, 13].

In addition, ratios between lipoproteins and HDL-C have also been suggested to better mirror the balance between pro- and antiatherogenic components in serum, and to add value in CVD risk estimation, particularly for patients with MetS [14]. In fact, the HDL-C ratios of triglycerides (TG/HDL-C) have been suggested as a biomarker of MetS in both females and males [15].

Furthermore, trimethylamine-N-Oxide (TMAO), a metabolite originating from microbial metabolism in the gut but also from the diet, has been shown to be associated with cardiovascular events and also to have direct pro-atherosclerotic properties [16]. Moreover, plasma TMAO levels has been found to be an independent risk factor for CVD-associated mortality in MetS patients [17]. The gut derived metabolites betaine and choline have also been suggested as biomarkers for CVD [16, 18].

Several studies have shown fluctuations in lipids and other cardiometabolic markers, during the menstrual cycle, reflecting the influence of both sex and gender [19–23]. Interestingly, in a previous study on fertile women, acute myocardial infarction was reported to be more common during the follicular phase [24]. However, most studies of gut microbiota and their role in CVD have been performed in males and there is to our knowledge no published studies on menstrual cycle variability of these biomarkers in humans [16]. Finally, in order to evaluate the utility of novel biomarkers, studies are ideally performed in healthy volunteers prior to investigations in patient populations [25].

Thus, the primary aim of this pilot-study was to evaluate variations in levels and ratios of non-HDL-C, ApoB, remnant-C and in gut microbiota TMAO, betaine and choline, in healthy volunteers during two menstrual phases. The secondary aim was to investigate possible correlations between these markers and estrogen and progesterone levels, and the correlation between the biomarkers for lipids and microbial metabolites.

## Methods

### Study population

Serum samples were retrieved from a recently performed prospective cohort study of 17 healthy women with regular menses [26, 27]. The original study was aimed at investigating menstrual cycle variability in serum and urinary concentrations in pharmacological biomarkers of CYP3A-activity and of biomarkers included in the athlete biological passport (ABP). In addition, the lipid profile in the different menstrual phases in relation to serum sex hormones levels was also studied. The power calculations were based on the number of subjects needed to show an association between menstrual cycle phases and the urinary epitestosterone used in ABP. Finally, the investigation was performed at the outpatient clinic at the Department of Clinical Pharmacology, Karolinska Hospital, Stockholm, Sweden.

In summary, females aged 18–45 years with regular menses as defined by cycle length 24–38 days, with vaginal bleeding 4–8 days and cycle variation ≤ 7–9 days were included [28]. Nineteen subjects were included in October and November 2018 after written informed consent and control of eligibility criteria. Two subjects later withdrew their consent due to lack of time to participate. Unfortunately, we were not able to recruit the 30 subjects needed for a 99% power as described above.

Subjects using oral contraceptives, who were breastfeeding or who were pregnant during the last three months were excluded. A physical examination was performed by a physician, including measurement of body mass index (BMI) and blood pressure.

No drugs containing hormones or drugs otherwise affecting the endocrine system were allowed. Over the counter vitamin supplements, and occasional Non-Steroidal Anti-inflammatory Drugs (NSAID) and paracetamol during the menstrual period were allowed. Other regular medication was allowed as judged by the investigator.

Blood samples in total 14 ml (one 4 ml EDTA and two 5 ml serum tube) were collected once a week during two menstrual cycles. Using this approach, sampling would capture the menstrual phase the subject was in at the time of investigation, based on an algorithm. The sampling procedure was done between 7 and 10 AM, and subjects were in a non-fasting state in order to reflect the atherogenic lipid profile in subjects over a 24-h period. Depending on cycle length, every subject had between six to nine blood tests. Subjects monitored their bleeding pattern throughout the two cycles with an app or on paper.

### Laboratory analyses

Routine safety parameters-hemoglobin, blood cells, c-reactive protein (CRP), albumin and creatinine were analyzed by the Laboratory of Clinical Chemistry, Karolinska University Hospital, Solna Sweden with ISO 15189:2012 accredited methods. The Karolinska Laboratory Medical Study Centre, a facility performing analysis of research samples according to Good Clinical Practice (GCP) standards, conducted analysis of the remaining blood samples. Serum was separated by centrifugation and stored at − 80 °C until analysis.

Progesterone, and estrogen were determined by radioimmunoassay while luteinizing hormone (LH) and follicle stimulating hormone (FSH) were determined using electrochemiluminescence immunoassay. TG, total cholesterol (TC) and HDL-C were analyzed by enzymatic assay followed by photometry. Apolipoprotein A1 (ApoA1) and ApoB were analyzed by immunochemistry followed by turbidimetry. LDL-C was calculated according to Friedewald, Levy and Fredrickson [29] while non-HDL-C and remnant-C were calculated as described by Carr et al. [10].

### Analysis of TMAO, betaine and choline

Analysis of TMAO, choline and betaine in serum samples were performed with LC–MS/MS at Swedish Metabolomic Centre in Umeå, Sweden using a protocol previously described [30].

### Definition of menstrual cycle phases

The participants phase in the menstrual cycle at the time of each blood sampling was established using the algorithm:

Follicular phase defined as estrogen < 81 pg/mL, progesterone < 1.6 ng/mL and FSH and LH.

Ovulatory phase defined as estrogen ≥ 81 pg/mL, progesterone < 1.6 ng/mL and LH higher than FSH. Finally, luteal phase defined as progesterone > 5.3 ng/mL [31].

### Statistical analysis

All analyses were done in Stata 15 (Stata Corp 2017. Stata Statistical Software: Release 15. College Station, TX: StataCorp LLC.) The comparisons between different phases of the menstrual cycle were done with linear regression, sub-setting the data to include two phases at the time. The dependencies between different variables were also investigated with linear regression. Finally, the cluster robust covariance estimator was used in all regression analysis to account for the inter-person correlation caused by repeated measurements. In this manner we adjust for some subjects having two complete menstrual cycles and some only one.

## Results

Of the 17 participants, 13 had two complete menstrual cycles, including the three phases follicular, ovulatory and luteal, according to the hormone levels measured in the samples. Thus, four participants contributed with only one complete menstrual cycle, including the three phases. Although data from the follicular and luteal phases were available for two cycles in these four subjects, the sampling had failed to capture the short ovulatory phase, as it lasts only 24 h.

None of the subjects were smokers, and none used other forms of tobacco. Six subjects did not use alcohol, and eleven subjects had low alcohol intake once or twice per week. Fourteen subjects exercised between two to seven days per week (mean 3.3 days per week), while three exercised once a week.

Mean baseline values for the subjects at inclusion were age 33.2 years, BMI 22.0 kg/m^2^, blood pressure 114/76 mm Hg and pulse 76 beats per minute. During the study, mean bleeding days were 7 and the mean cycle length was 24.5 days. There were no significant changes in safety laboratory parameters during the study.

In total, blood samples from 30 complete menstrual cycles, including follicular, luteal and ovarian phases, could be retrieved from 17 women. Thus, 90 blood samples were analyzed for sex-hormones, lipids, TMAO, betaine and choline.

### Sex hormones

**Fig. 1:**
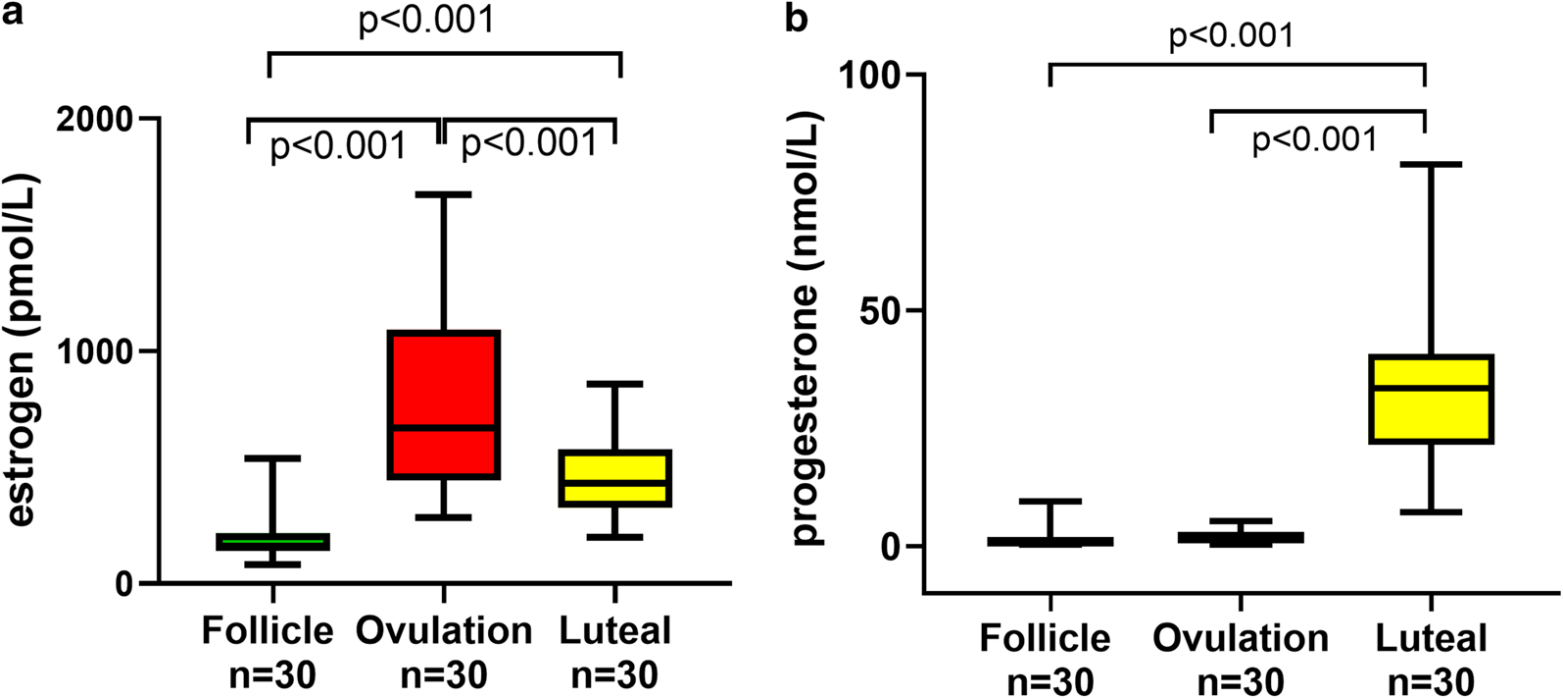
**a**, **b** Variations in estrogen and progesterone throughout the different phases in 30 menstrual cycles from 17 fertile women

To confirm known variations in sex hormones during the menstrual cycle, levels of estrogen and progesterone, as well as FSH and LH, were controlled. For estrogen there were significant differences between all phases (Fig. 1a), whereas progesterone was significantly higher in the luteal phase (Fig. 1b).

### Lipids and lipoproteins

**Fig. 2:**
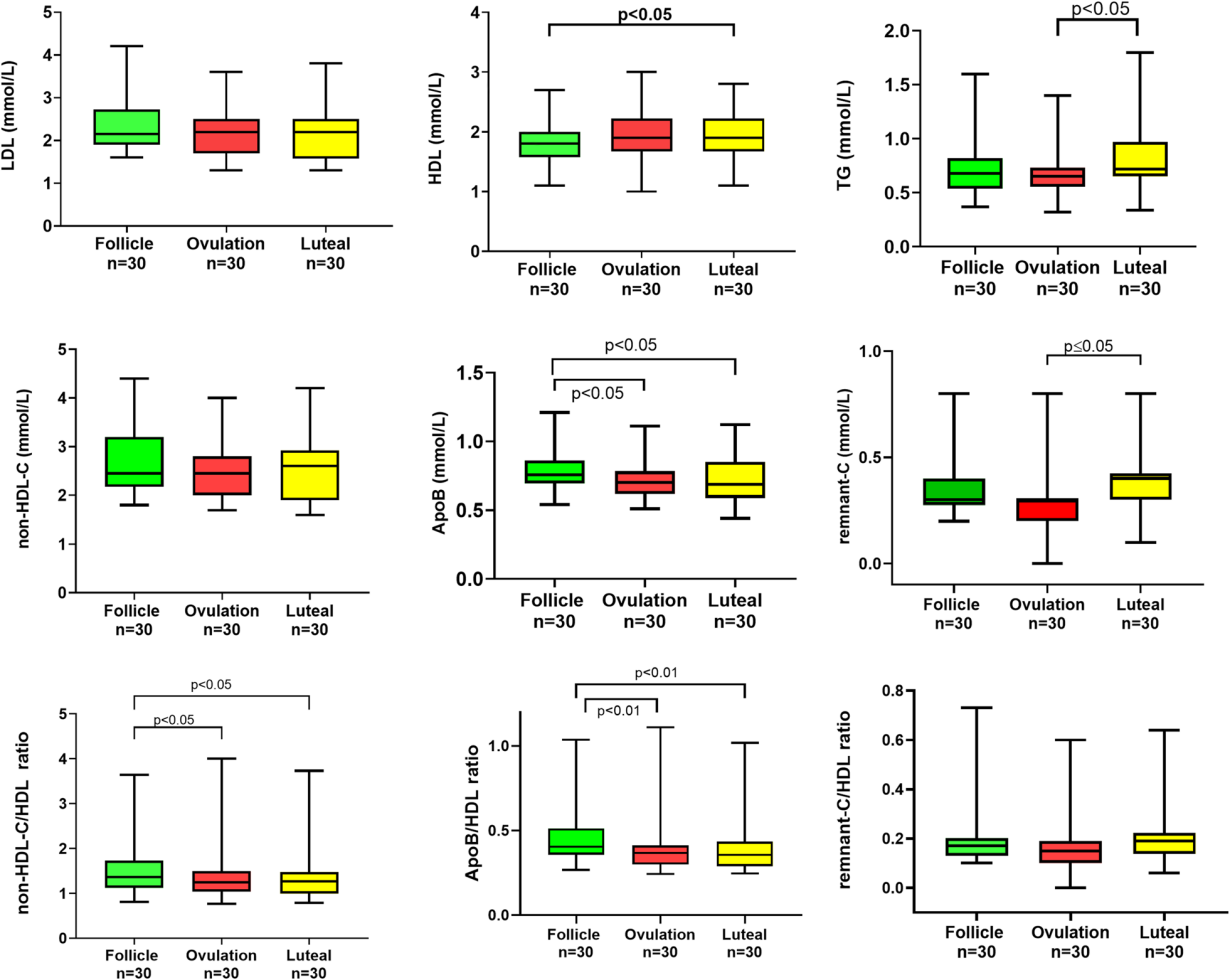
Variations in Low-Density Lipoprotein-Cholesterol (LDL-C), High-Density Lipoprotein-Cholesterol (HDL-C), Triglycerides (TG), non-High-Density Lipoprotein-Cholesterol (non-HDL-C), remnant-Cholesterol (remnant-C) and Apolipoprotein B (ApoB) levels and their HDL-ratios throughout follicular, ovulatory and luteal phases of 30 menstrual cycles from 17 fertile women

Levels of HDL-C and TG were higher during the luteal phase when estrogen levels are higher (Fig. 2). Levels of the new biomarkers ApoB, ApoB/HDL and non-HDL-C/HDL ratio were highest during the follicular phase, while remnant-C levels were highest during the luteal phase (Fig. 2).

**Fig. 3:**
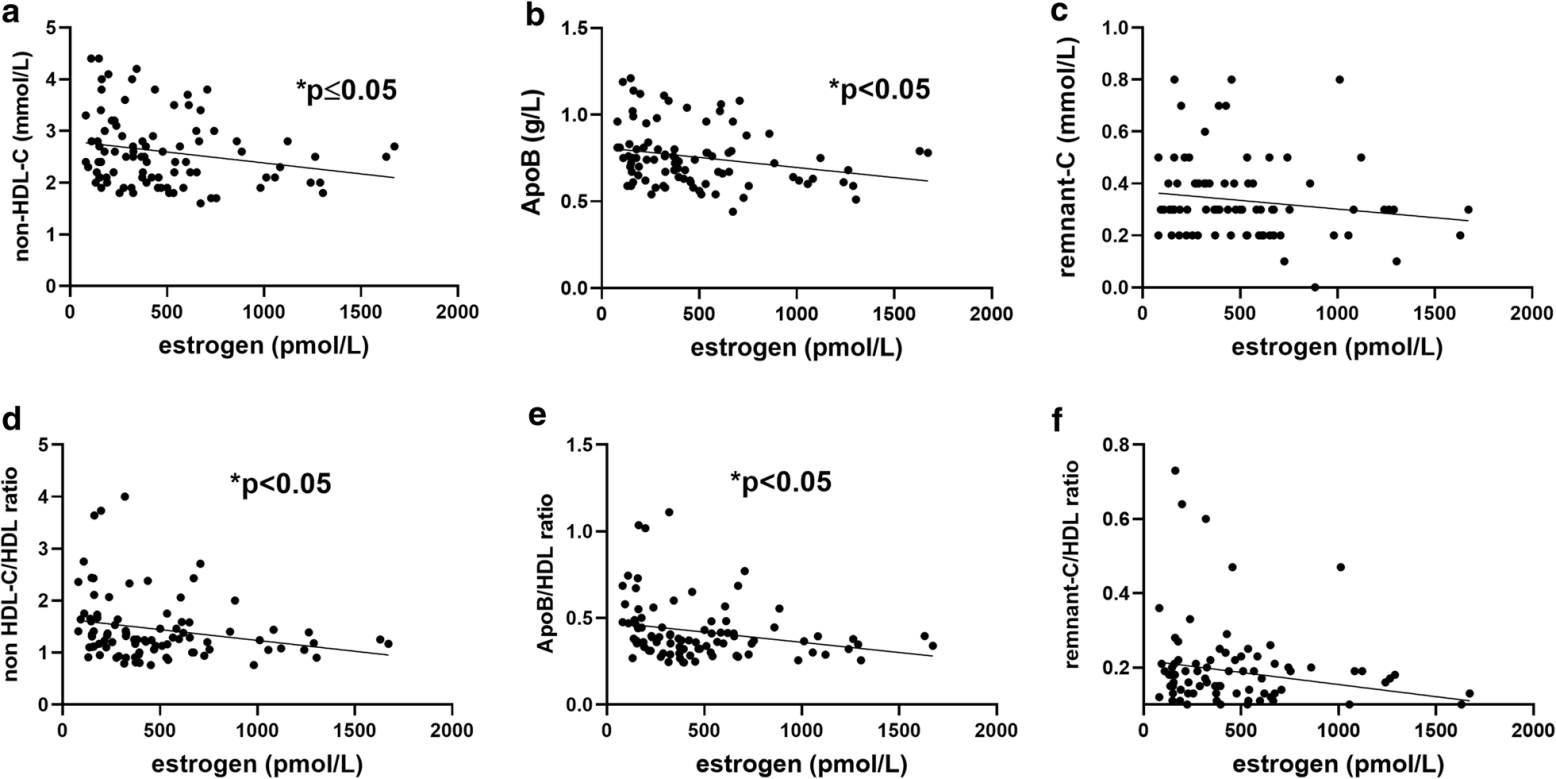
Correlation analysis between estrogen levels and biomarkers for dysplipidemia: **a** non-High-Density Lipoprotein-Cholesterol (non-HDL-C), **b** Apolipoprotein B (ApoB), **c** remnant-Cholesterol (remnant-C) and **d**–**f** their HDL-ratios throughout follicular, ovulatory and luteal phases of 30 menstrual cycles from 17 fertile women

Low levels of estrogen were associated with higher levels of non-HDL-C and ApoB (Fig. 3). Likewise, low levels of estrogen were associated with higher levels of the lipid ratios non-HDL-C/HDL, and ApoB/HDL (Fig. 3).

There was no correlation between estrogen and remnant-C or the remnant -C/HDL ratio (Fig. 3). No correlation was found between any of the lipids, lipoproteins or ratios and progesterone levels.

To summarize, higher levels of pro-atherogenic lipoproteins and their HDL-ratios was observed during the follicular phase, while remnant-C increased during the luteal phase. Significant correlation between the ratios for dyslipidemia and estrogen was shown, with higher levels of pro-atherogenic biomarkers associated with low estrogen levels.

### TMAO, betaine and choline

**Fig. 4:**
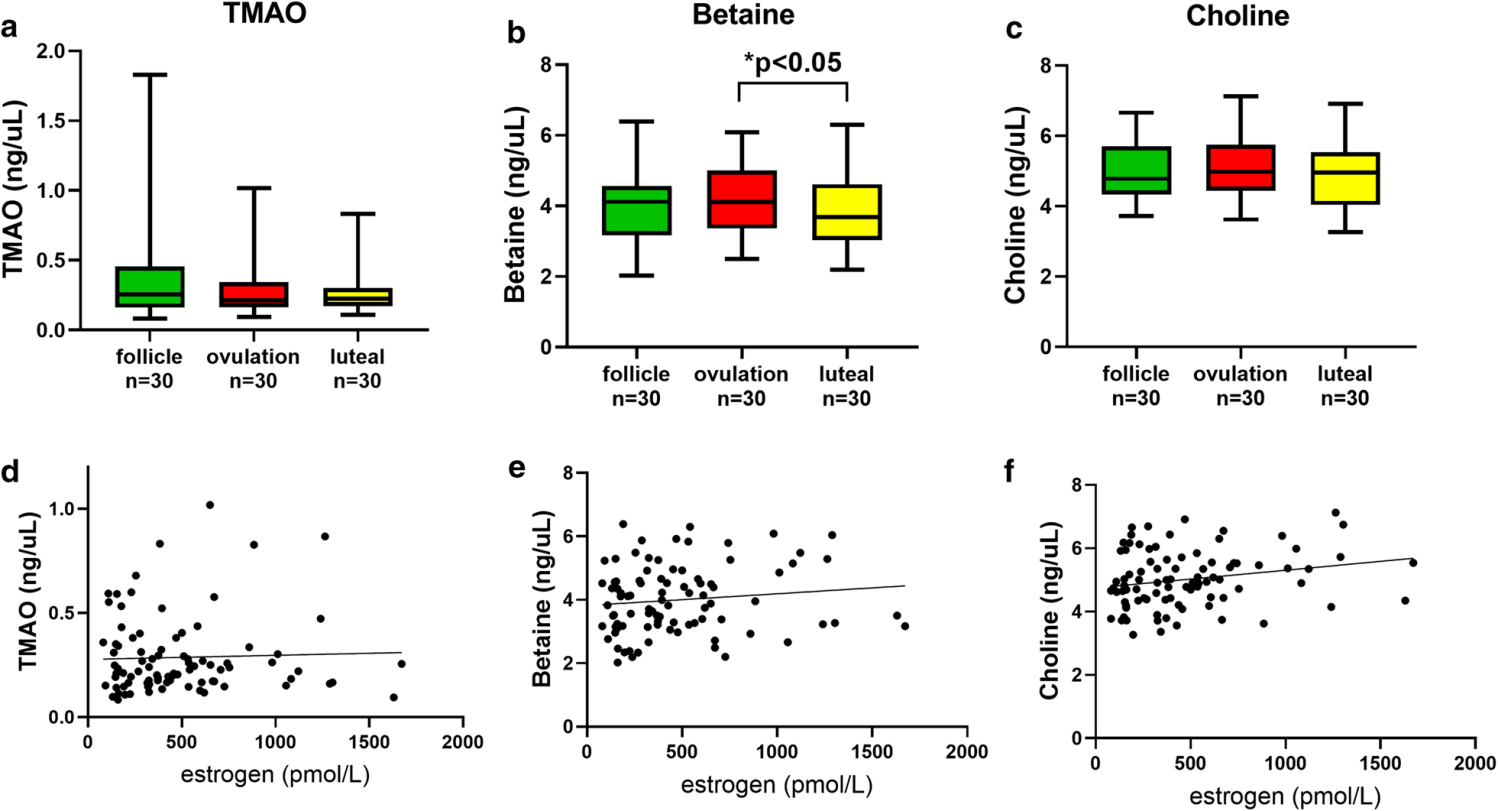
Variations in levels of gut microbiota metabolites **a** trimethylamine-N-Oxide (TMAO), **b** betaine and **c** choline  in plasma throughout the different phases in 30 menstrual cycles from 17 fertile women and **d**–**f** shows correlation analysis between the metabolites and estrogen (n = 90)

There were no significant differences in TMAO and Choline levels during follicular, ovulatory and luteal phases. In contrast, betaine levels were higher in the ovulatory phase compared to the luteal phase (*p* < 0.05) (Table 1, Fig. 4). None of the microbiota metabolites showed any correlation with estrogen or with progesterone levels.

**Table 1:** Plasma levels of novel biomarkers for dysplipidemia and dysbiosis during the follicular, ovulatory and luteal phase of 30 menstrual cycles in 17 regularly menstruating women

Parameter	Follicular phase Mean ± SD Median	Ovulatory phase Mean ± SD Median	Luteal phase Mean ± SD Median
TMAO (ng/µL)	0.35 (± 0.33) 0.25	0.31 (± 0.24) 0.21	0.25 (± 0.14) 0.22
Betaine (ng/µL)	3.99 (± 10.7) 4.11	4.20 (± 1.01) 4.13	3.81 (± 1.12) 3.67
Choline (ng/µL)	4.98 (± 0.85) 4.79	5.15 (± 0.87) 4.98	4.93 (± 1.00) 4.96
Non-HDL-C (mmol/L)	2.7 (± 0.72) 2.45	2.51 (± 0.64) 2.45	2.60 (± 0.71) 2.60
Remnant-C (mmol/L)	0.33 (± 0.12) 0.30	0.30 (± 0.15) 0.30	0.38 (± 0.17) 0.40
ApoB (g/L)	0.80 (± 0.17) 0.75	0.73 (± 0.16) 0.70	0.74 (± 0.18) 0.68
Non-HDL-C/HDL	1.56 (± 0.63) 1.36	1.40 (± 0.67) 1.24	1.40 (± 0.59) 1.27
ApoB/HDL	0.46 (± 0.17) 0.40	0.41 (± 0.18) 0.38	0.40 (± 0.15) 0.36
Remnant-C/HDL	0.19 (± 0.12) 0.17	0.17 (± 0.12) 0.15	0.21 (± 0.12) 0.19

TMAO, trimethylamine N-oxide; non-HDL-C, non-High-Density Lipoprotein-Cholesterol; remnant-C, remnant-Cholesterol; ApoB, Apolipoprotein B

### Correlation between TMAO, choline, betaine and non-HDL-C, remnant-C and ApoB levels

**Fig. 5:**
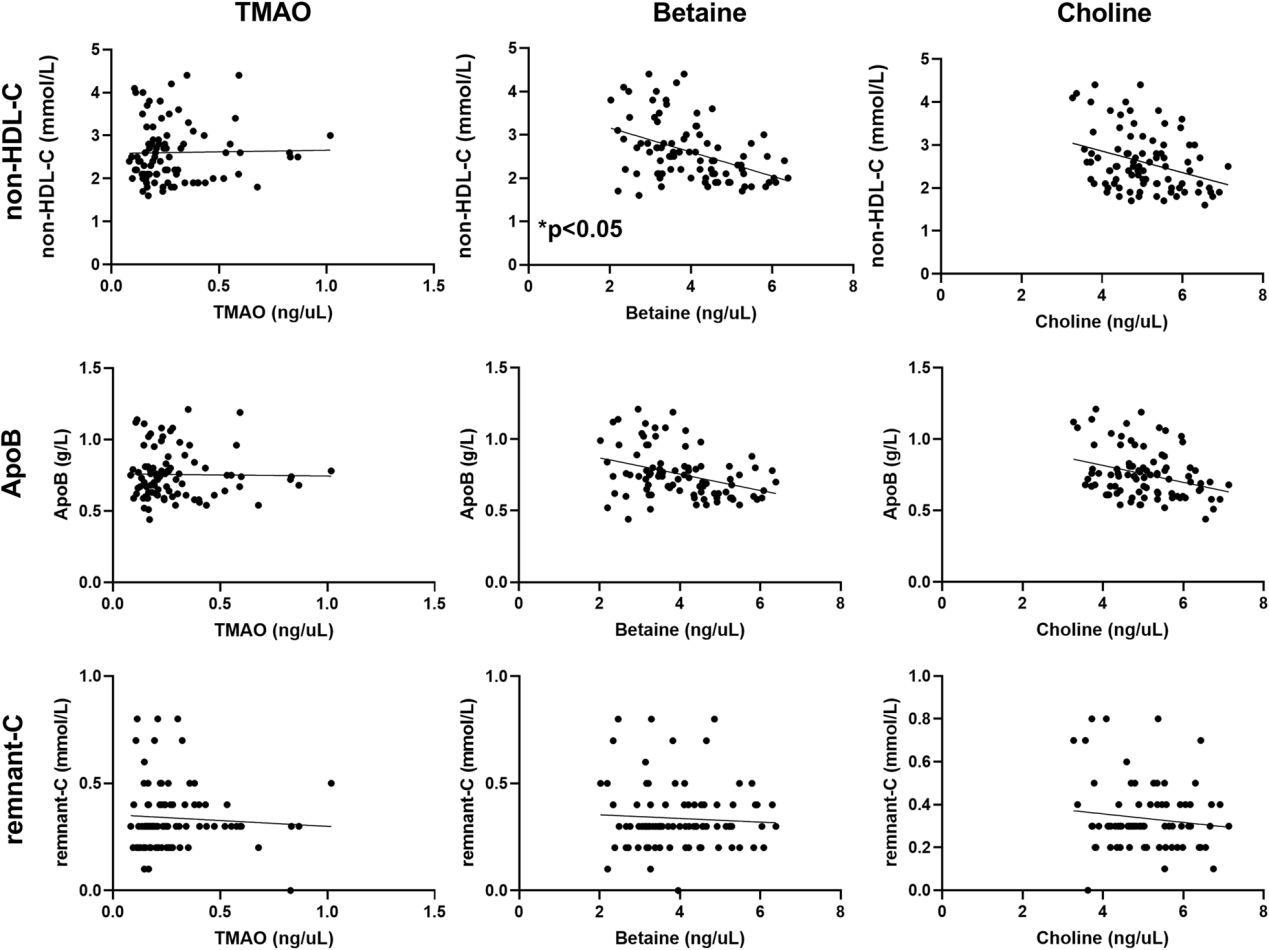
Correlation analysis of non-High-Density Lipoprotein-Cholesterol (non-HDL-C), Apolipoprotein B (ApoB) and remnant-Cholesterol (remnant-C) and gut microbiota metabolites trimethylamine-N-Oxide (TMAO), choline and betaine throughout follicular, ovulatory and luteal phases of 30 menstrual cycles from 17 fertile women (n = 90)

There were no significant correlations between TMAO or choline and lipids or lipid ratios (Fig. 5). There was a weak association between betaine and non-HDL-C; *p* < 0.05 (Fig. 5). To conclude, no strong association between the gut microbiota metabolites and the new lipid biomarkers were observed.

## Discussion

In this study, we show that low estrogen levels, as present during the follicular phase of the menstrual cycle, was associated with higher levels of proatherogenic non-HDL-C, and ApoB and their HDL-ratios. Remnant-C was higher during the luteal phase and showed no correlation with estrogen levels. The pro-atherogenic microbial metabolite TMAO did not show any association with levels of sex hormones or lipid biomarkers.

The results of our study on menstrual cycle variability in lipids and their ratios are in line with previously performed studies with higher levels of proatherogenic lipids during the follicular phase compared to luteal phase [20–22]. The HDL-C levels were higher during the luteal phase when estrogen is high [32]. The decrease in ApoB and ApoB/HDL ratio in the ovulatory and luteal phase of our study, has not been observed previously [33, 34]. However, this is in agreement with previous findings of decreased atherogenicity during the luteal phase [20].

The significant increase in remnant-C and its surrogate marker TG [13] during the luteal phase, could be due to the non-fasting sampling in the present study. However, as sampling in the other phases were performed in the same manner, this is not likely to be the cause. Another possible explanation is the increase in prevalence of high TG and MetS in the general population, compared to lipid studies performed 20 to 30 years ago. Mendelian randomization studies suggest that TG-rich lipoproteins like remnant-C are causally associated with an increased risk of CVD and to all-cause mortality, particularly in women [19, 35, 36]. The implications of our study could be that sampling for evaluating risk in patients with MetS and/or high TG, may be performed not only during the follicular phase but also during the luteal phase. If remnant-C/HDL ratio is evaluated, sampling during the follicular phase could be sufficient. However, there are no established reference values for the ratios non-HDL-C/HDL, ApoB/HDL and remnant-C/HDL in adults, as opposed to age- and/or sex-specific values for levels of non-HDL-C, remnant-C and ApoB respectively [37].

It has been suggested that a combination of biomarkers will improve the CVD assessment and the newly added biomarkers give different contributions to such an evaluation. Non-HDL-C includes Lp(a), a more powerful predictor of CVD incidence in women compared to men [8]. Non-HDL-C also confer information on total cholesterol content, measured as mass. ApoB is a measure of the number of atherogenic particles in the circulation [10] while remnant-C provides information on the cholesterol content in the postprandial triglyceride-rich lipoproteins (TGRLs) [13].

The results from our study do not support a strong association between estrogen and the levels of the microbial metabolites TMAO, betaine or choline in blood. In a previous study of 25 males and 7 postmenopausal women, a strong association was found between levels of estrogens and their metabolites in urine and the diversity of microbiota in feces [38]. However, no association for premenopausal women was found in the study, which is concurring with our results. This could also be related to the sampling of the fertile women, being performed irrespective of menstrual cycle phase, resulting in highly variable estrogen levels. Interestingly, in a review studies of fecal microbiota transplant studies to patients with MetS and obesity, none of the 76 included patients were female [39].

Notably, no correlation between TMAO and the new lipid biomarkers were observed in this study. Still, TMAO appears to play an important role in cholesterol metabolism, causing inhibition of reverse cholesterol transport and an increase in foam cell activity by activating macrophages in the immune system [16]. In addition, TMAO may repress bile acid synthesis, thus effecting the major pathway for cholesterol elimination [16]. TMAO homeostasis is complex and is not only affected by gut microbiota but also by e.g. diet, age, gender, BMI and kidney function [40].

The link between the gut microbiota and CVD has become stronger in recent years [40]. However, TMAO is still not validated for clinical use in CVD risk assessment and a study of healthy middle-aged adults, did not find any association between TMAO and the risk of advancing atherosclerosis [41].

The strength of our study is that the exact menstrual phases were verified by measuring FSH, LH, estrogen and progesterone on a weekly basis. Also, lipids were measured at the same time point as sex hormones. Furthermore, a novelty in our study was that the sampling was performed in a non-fasting state in accordance with recent consensus [12] and in contrast with most previously performed studies [22]. In this manner, triglyceride-rich lipoproteins were also included in the lipid profile. Not controlling for dietary intake, also increases the probability of having a dietary status more representative of ordinary life. Data on relevant lifestyle factors like physical activity, smoking/snuff and alcohol intake that may affect lipid homeostasis, were also registered. This study is also the first describing the levels of gut microbiota metabolites in the circulation in healthy, fertile women in the different menstrual cycle phases.

However, a major limitation in this study is the small sample size. Thus, the findings should be confirmed in a future study with a larger sample size. In addition, standardization of diet could be considered in order to evaluate whether dietary intake of choline and betaine affect our findings between these markers and lipids [18]. Finally, the use of calculated LDL and remnant-C may introduce error sources. There are currently no commercially available direct assays method for remnant-C, but there is for LDL, although not commonly used in routine clinical practice [12].

The next step would be to investigate the biomarkers for dyslipidemia and dysbiosis in a patient cohort with MetS, but without polycystic ovary syndrome, during the menstrual cycle. Further elucidating the connections between hormones, microbiota and dyslipidemia may contribute to better treatment options for women, both pharmacological and non-pharmacological.

In recent years it has been suggested that B-vitamins and omega-3-polyunsaturated fatty acids may affect dyslipidemia and dysbiosis [42, 43]. In future studies, performed on women with metabolic syndrome or other risk factor for CVD, it would be interesting to study the interplay between these nutrients and the biomarkers studied here.

Vitamin D has been shown to affect endometrial function and low levels of vitamin D is associated with impaired fertility and Polycystic Ovary Syndrome (PCOS) [44, 45]. In addition, Vitamin D deficiency has been shown to be associated with high levels of TMAO and obesity [46]. In future studies it would be interesting to study the relationship between Vitamin D and TMAO in women with PCOS and if it affects the risk of CVD. The results from the present study, including healthy, fertile women without PCOS, could constitute a valuable reference cohort for such studies.

## Conclusion

For the first time, we have analyzed the levels of different microbial metabolites in blood during the menstrual cycle. Furthermore, our data supports that the new biomarkers recommended for estimation of CVD-risk and as treatment targets in dyslipidemia, vary during the menstrual cycle.

The increased prevalence in obesity, metabolic syndrome and CVD in fertile, menstruating women, indicate that in years to come, there will be a high need for new, additional biomarkers in combination to optimize CVD assessments and treatment in this population. Further studies of menstrual cycle variability in fertile women with metabolic syndrome/type 2 diabetes and previous CVD, is warranted.

## Data Availability

As sample size is small, there is a risk of the individual privacy being compromised. De-identified data are therefore available from the corresponding author upon reasonable request.

## Abbreviations

CVD: Cardiovascular disease
FSH: Follicle stimulating hormone
LH: Luteinizing hormone
LDL-C: Low-Density Lipoprotein-Cholesterol
HDL-C: High-Density Lipoprotein-Cholesterol
Non-HDL-C: Non-HDL cholesterol
TC: Total Cholesterol
ApoB: Apolipoprotein B
ApoA1: Apolipoprotein A1
Remnant-C: Remnant-Cholesterol
TGRLs: Triglyceride-Rich Lipoproteins
VLDL: Very Low-Density Lipoprotein
IDL: Intermediate-Density Lipoprotein
TMAO: Trimethylamine-N-Oxide
MetS: Metabolic syndrome
AMI: Acute myocardial infarction
ESC: European Society of Cardiology
EAS: European Society of Atherosclerosis
ABP: Athletes biological passport
CRP: C-reactive protein
BMI: Body mass index
NSAID: Non-steroidal anti-inflammatory drug
GCP: Good Clinical Practice
PCOS: Polycystic Ovary Syndrome
